# Fournier’s Gangrene and Extensive Abdominal Necrotizing Fasciitis Secondary to Perforated Appendicitis Within an Inguinoscrotal Hernia: A Rare Case Report

**DOI:** 10.7759/cureus.114723

**Published:** 2026-08-18

**Authors:** Mohammed Ramdani, Anouar El Moudane, Youness Tahri, Oussama Slimani, Ali Barki

**Affiliations:** 1 Urology, Mohammed VI University Hospital Oujda, Oujda, MAR; 2 Urology, Faculty of Medicine and Pharmacy of Oujda, Oujda, MAR; 3 Urology, Urology, Mohammed VI University Hospital Oujda, Oujda, MAR; 4 Radiation Oncology, Faculty of Medicine and Pharmacy, Mohammed First University, Oujda, MAR; 5 Urology, Mohammed VI University Medical Center, Faculty of Medicine and Pharmacy, Mohammed the First University, Oujda, MAR

**Keywords:** fournier's gangrene, hyperbaric oxygen therapy (hbot), invasive surgical debridement, necrotizing fasciitis, perforated appendicitis –, polymicrobial infection

## Abstract

Fournier's gangrene (FG) is a life-threatening necrotizing infection rarely triggered by an intra-abdominal process. We report an unusual case of FG and extensive abdominal necrotizing fasciitis secondary to a perforated appendix within an inguinoscrotal hernia. A 66-year-old male presented with a three-day history of progressive abdominal pain and systemic toxicity. Physical examination revealed abdominal contracture, extensive erythema, subcutaneous emphysema of the lower abdomen, and necrotic patches on the right scrotal skin. A computed tomography (CT) scan confirmed FG secondary to acute perforated appendicitis. The patient underwent emergency surgery via a combined midline laparotomy and scrotal approach, revealing purulent peritonitis and widespread tissue necrosis. Wide surgical excision and thorough debridement were performed. Postoperative management included broad-spectrum antibiotics and adjuvant hyperbaric oxygen therapy (HBOT). Tissue cultures grew a polymicrobial infection (Escherichia coli, Streptococcus spp., Pseudomonas aeruginosa, and Bacillus). Following intensive multidisciplinary care and sequential debridements, the patient's condition successfully stabilized. This case highlights the significance of recognizing Fournier's gangrene as a potential complication of an intra-abdominal septic process.

## Introduction

Fournier's gangrene is a rare, life-threatening necrotizing soft tissue infection primarily affecting the genital, perineal, and perianal regions [1,2]. While its etiology is most commonly linked to primary colorectal or urogenital sources, it can occasionally arise as a severe complication of acute appendicitis [1-3]. Appendicitis typically progresses to localized or generalized peritonitis when left untreated; however, in exceptional cases, the spread of infection extends further to involve the perineal tissue planes, culminating in Fournier's gangrene [3].

When this rare complication occurs, patients present with a combination of acute abdominal pain and systemic toxicity, accompanied by localized erythema, severe pain, and tissue necrosis [1-3]. Due to the rapid progression toward sepsis, multi-organ failure, and death, prompt clinical recognition is critical [1,2]. Diagnosis relies heavily on thorough physical examination, and optimal management requires immediate surgical debridement alongside broad-spectrum antibiotic coverage and intensive supportive care [1,2].

Given the extreme scarcity of appendiceal peritonitis as a trigger for Fournier's gangrene, documented almost exclusively in sporadic case reports [3], further characterization of this entity is essential. Here, we present the case of a 66-year-old male presenting with a three-day history of severe abdominal pain who was diagnosed with Fournier's gangrene secondary to appendicular peritonitis, followed by a discussion of its diagnostic and therapeutic challenges.

## Case presentation

A 66-year-old man sought emergency medical care for abdominal pain that had been worsening over the preceding three days, associated with a deterioration of his general status, anorexia, and acute scrotal pain radiating to the pubic region. His medical history was significant for type 2 diabetes managed with oral antidiabetics and a right radical nephrectomy in 2015 for a renal tumor.

**Figure 1:**
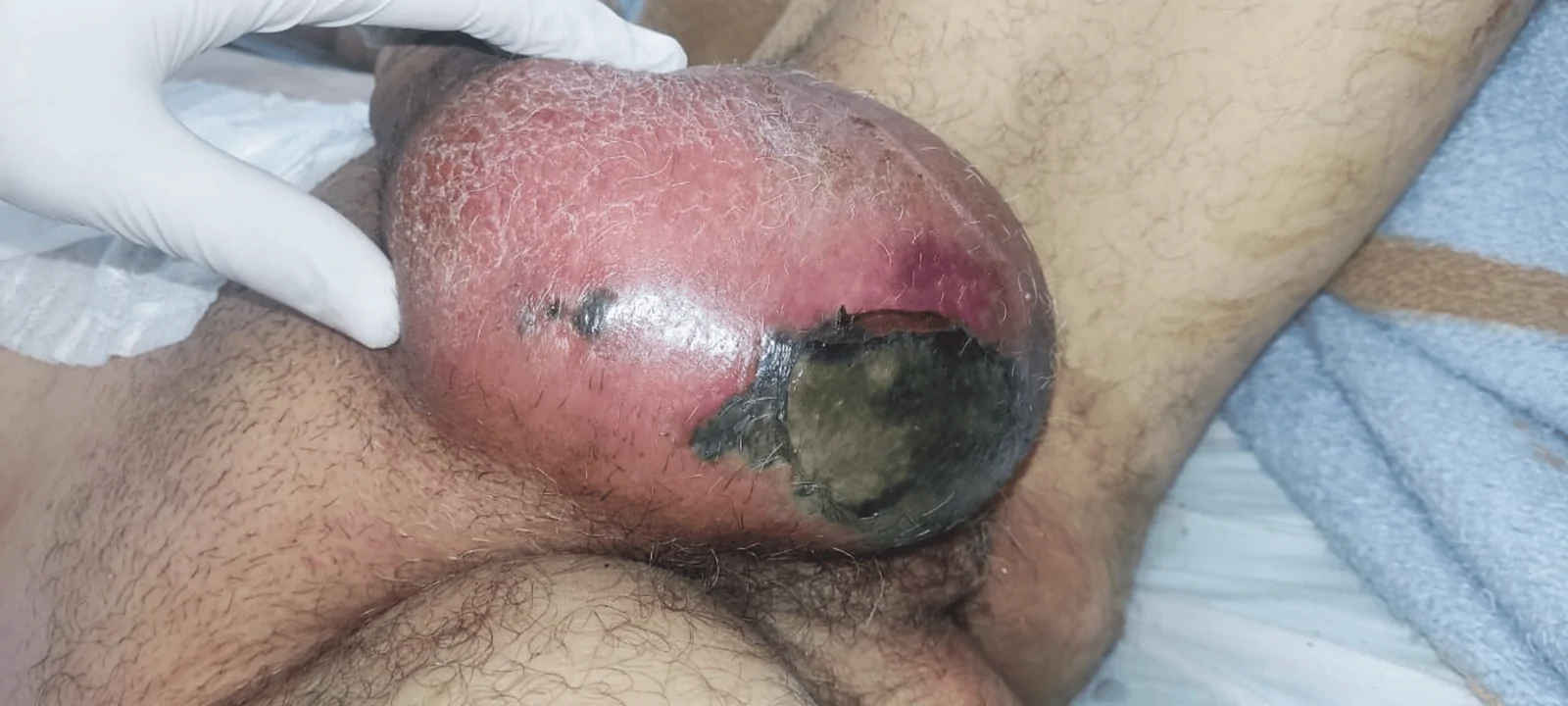
Clinical image showing scrotal inflammatory signs, infiltration of the scrotal skin, and necrosis in the right scrotal.

Upon physical examination, the patient was tachycardic with a fever of 39.3°C. He exhibited clinical signs of dehydration and sepsis. Abdominal examination revealed guarding and contracture. Furthermore, extensive erythema and subcutaneous emphysema were noted across the perineum and the abdominal wall. Examination of the external genitalia revealed significant scrotal swelling with inflammatory changes, skin infiltration, and focal necrosis of the right scrotal area (Figure 1).

Initial blood tests revealed a white blood cell count of 13,310/mm³, severe anemia with a hemoglobin level of 7.9 g/dL, and a markedly elevated C-reactive protein (CRP) concentration of 446 mg/L. Electrolyte and renal panel showed a serum sodium level of 134 mmol/L, potassium of 4.7 mmol/L, and serum creatinine of 14 mg/L (1.4 mg/dL), while liver function parameters remained within normal limits. Based on these admission laboratory values, the patient's LRINEC score was 8 (indicating a high risk of necrotizing soft tissue infection), and the initial Fournier’s Gangrene Severity Index (FGSI) score was 7 (predicting a high probability of survival).

**Figure 2:**
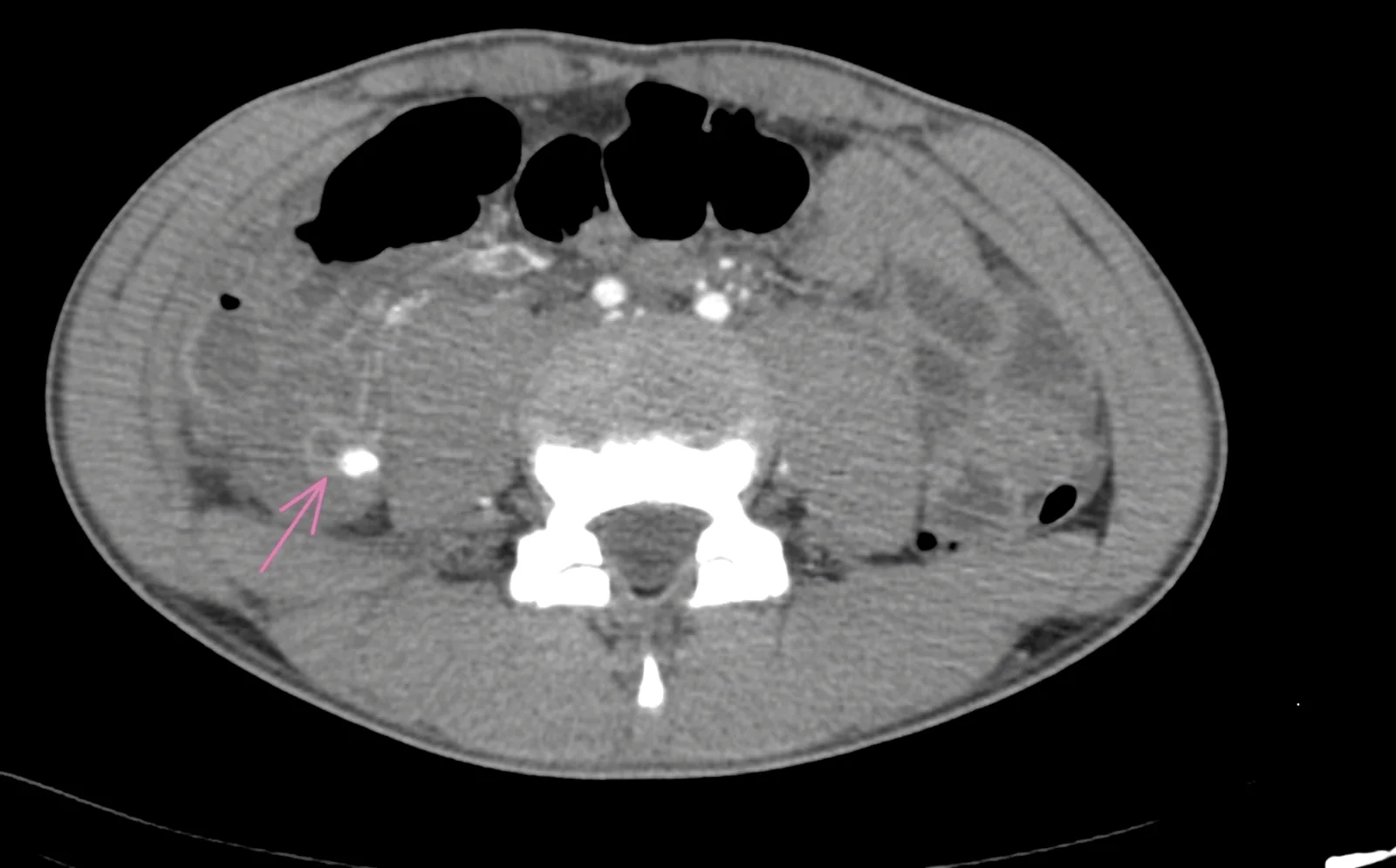
Scannographic image showing the swollen appendix, with enhanced wall after injection, containing a fecalith associated with intra-peritoneal fluid collection suggestive of appendicular peritonitis.

An abdomino-pelvic CT scan revealed findings consistent with perforated appendicitis and localized peritonitis. Notably, it also identified a right inguino-scrotal hernia (35 mm neck) containing omental (epiploic) content. The scan further demonstrated gas-forming necrotizing infection of the right anterolateral abdominal wall, dissecting the subcutaneous tissues from the right iliac fossa down to the scrotal region (Figure 2).

The patient was immediately transferred to the operating theater for an urgent laparotomy. A midline incision confirmed a perforated appendix with purulent peritonitis. Surgical exploration revealed extensive necrotizing fasciitis involving the fascial and muscular layers of the right lower abdominal wall, extending from the right iliac fossa to the inguinoscrotal region, with retroperitoneal involvement reaching the previous right nephrectomy bed. A scrotal exploration was performed to assess testicular viability. As the testis was viable and showed no evidence of ischemia or necrosis, orchiectomy was not indicated, and the testis was preserved. Extensive debridement of all necrotic tissues was carried out until healthy, bleeding tissue margins were obtained, followed by copious irrigation. Owing to the extent of soft-tissue loss and the need for repeated reassessment, the wound was intentionally left open.

Following surgery, the patient was admitted to the surgical intensive care unit (ICU). His postoperative course was complicated by transient hemodynamic instability and disseminated intravascular coagulation (DIC), requiring low-dose dopamine support and transfusion of 3 units of packed red blood cells and 15 units of fresh frozen plasma. Empirical broad-spectrum antimicrobial therapy, consisting of a third-generation cephalosporin, metronidazole, and gentamicin, was initiated immediately and combined with adjunctive hyperbaric oxygen therapy.

A planned second-look debridement was performed 48 hours after the initial operation. Thereafter, the patient underwent more than four serial surgical debridements, according to daily clinical assessment of the wound. Daily dressing changes were performed, and any newly identified devitalized tissue was excised to ensure complete infection control. Negative-pressure wound therapy (VAC) was not used because this modality was not available at our institution.

Blood cultures grew Escherichia coli. Microbiological cultures of the debrided tissue demonstrated a polymicrobial infection with Escherichia coli, Streptococcus spp., Pseudomonas aeruginosa, and Bacillus spp. Progressive clinical improvement was achieved with repeated surgical debridement, intensive wound care, targeted supportive therapy, and antimicrobial treatment. After complete control of the infection and the development of a healthy granulating wound bed, definitive soft-tissue reconstruction was performed using split-thickness skin grafting. The patient was discharged 40 days after admission. At the one-year follow-up, complete wound healing had been achieved, with no evidence of recurrent infection or functional impairment.

## Discussion

Fournier's gangrene (FG) is a rare yet serious infection of the soft tissue in the genital, perineal, and perianal areas. The condition is characterized by tissue death and can be caused by a breach in the gastrointestinal or urethral mucosa. It can affect individuals of any gender or age and is considered life-threatening. Fournier's gangrene causes thrombosis of small blood vessels, leading to an obliterative endarteritis, which ultimately results in necrosis of the skin and surrounding tissues [1].

The development of Fournier's gangrene (FG) is linked to a variety of both modifiable and non-modifiable risk factors [2]. Modifiable determinants represent variables within a patient's control to change, whether through therapeutic interventions or lifestyle adjustments. This classification comprises chronic conditions such as diabetes and substance abuse, among others. In contrast, non-modifiable elements, including a patient's age, are inherently unalterable. Existing literature indicates that a significant majority of affected individuals present with pre-existing medical conditions, notably diabetes, intravenous drug use, hepatic failure, and compromised immune function.

Acute appendicitis is a common surgical emergency, affecting approximately 7% of the population during their lifetime. Although it primarily affects young adults, its incidence is increasing among elderly patients. Necrotizing fasciitis secondary to acute appendicitis remains exceptionally rare but is associated with a fulminant clinical course and high mortality, particularly in elderly or immunocompromised individuals [3]. In the present case, the development of Fournier's gangrene can be explained by the unusual anatomical location of the inflamed appendix within an inguinoscrotal hernia sac. When appendiceal inflammation progresses to gangrene or perforation, the hernia sac provides a direct pathway for enteric bacteria and inflammatory exudate to spread along the inguinal canal into the scrotal and perineal soft tissues. This contiguous extension bypasses the usual intraperitoneal dissemination observed in conventional perforated appendicitis and creates favorable conditions for the development of polymicrobial necrotizing fasciitis. Several published case reports have described Fournier's gangrene as a rare but devastating complication of perforated Amyand's hernia, highlighting the importance of early diagnosis and prompt surgical management. Therefore, in patients presenting with inguinoscrotal sepsis, subcutaneous emphysema, or Fournier's gangrene associated with an incarcerated inguinal hernia, clinicians should consider the possibility of an underlying perforated appendicitis within the hernia sac, as delayed recognition may significantly worsen the prognosis [4].

Microbiologically, isolated aerobic bacteria are detected in 10% of cases, whereas anaerobic strains are identified in approximately 20%. The predominant portion, accounting for the remaining 70%, presents with a mixed population of both aerobic and anaerobic organisms. Monobacterial growth is rare, with a single organism isolated in fewer than 10% of wound cultures. Furthermore, data from two extensive reviews reported a mean yield of 3.1 to 4.6 organisms per culture specimen. Frequently encountered aerobes include group A Streptococcus, Staphylococcus aureus, Escherichia coli, and other Enterobacteriaceae. The anaerobic spectrum routinely encompasses Peptostreptococcus spp., Prevotella spp., Porphyromonas spp., Fusobacterium spp., Bacteroides spp., and Clostridium spp [5].

The diagnosis of Fournier’s gangrene (FG) relies primarily on clinical evaluation. The disease frequently features an insidious onset, with up to 40% of patients initially presenting with vague or non-specific symptoms, which underscores the vital importance of early clinical suspicion [5,6]. Prior to the classic manifestations, prodromal signs such as fever and lethargy may persist for a few days. Initial localized symptoms typically involve pain within the genital or perianal regions, often occurring with minimal or absent visible cutaneous lesions. Subsequently, this discomfort intensifies in tandem with progressive erythema of the overlying skin. This is followed by a dusky discoloration of the integument and palpable subcutaneous crepitation. Ultimately, the infection culminates in overt gangrene, characterized by more definitive physical findings and purulent wound drainage. Notably, the testicles are frequently spared from the necrotic process due to their independent vascular supply derived from the internal spermatic arteries [6,7].

While diagnostic imaging provides valuable support in cases of suspected Fournier's gangrene, critical patients displaying hemodynamic instability and clear clinical signs should be fast-tracked directly to the operating suite for definitive surgical care, bypassing prior radiological assessment. It is essential to emphasize that normal imaging findings cannot eliminate the diagnosis, and diagnostic procedures must never delay operative treatment. Plain radiographs may demonstrate soft tissue edema and gas accumulation; however, the absence of subcutaneous air does not rule out the disease. Ultrasound offers superior utility compared to plain films for differential diagnosis and the examination of the testes and epididymides. Concurrently, computed tomography (CT) displays high sensitivity and specificity, serving as a powerful tool for diagnostic confirmation and surgical mapping. Frequent CT features include subcutaneous emphysema, irregular fascial thickening, abscess formation, and fluid loculations. Magnetic resonance imaging (MRI) is discouraged during the initial workup because of its high cost and prolonged scanning times, despite its exceptional soft-tissue resolution [7-9].

No dedicated biomarkers or specific laboratory assays exist for necrotizing soft tissue infections (NSTIs) or Fournier's gangrene (FG). Differentiation from alternative soft tissue infections cannot be reliably achieved through any single laboratory parameter due to insufficient sensitivity and specificity. Nevertheless, a combination of laboratory metrics allows clinicians to gauge disease severity and estimate mortality risks [7]. Crucial diagnostic variables include white blood cell count, C-reactive protein, hemoglobin, sodium, creatinine, and blood glucose. The standard diagnostic laboratory panel typically encompasses a complete blood count (CBC) with differential, culture of wound exudate or abscess fluid, a disseminated intravascular coagulation (DIC) profile, standard coagulation assays (e.g., PT, aPTT), fibrinogen and fibrin degradation product (FDP) measurements, concurrent blood and urine cultures, arterial blood gas (ABG) analysis, a serum electrolyte panel and renal function markers (BUN, creatinine), and blood glucose monitoring [10].

Several prognostic scoring indices are utilized to evaluate disease severity and forecast clinical progress. Notably, the Laboratory Risk Indicator for Necrotizing Fasciitis (LRINEC) score was designed by Wong et al. [1]. An LRINEC value of 8 or greater reflects a probability exceeding 75% for the presence of necrotizing fasciitis. This scoring method carries a 92% positive predictive value and a 96% negative predictive value [1]. In parallel, the Fournier Gangrene Severity Index (FGSI) grades nine clinical variables. An FGSI score greater than 10.5 correlates with a 96% risk of mortality, whereas a score equal to or below 10.5 indicates a 96% likelihood of survival. Both systems exhibit comparable performance in predicting patient prognosis and determining the necessity for intensive care admission [11,12]. In our case, the patient presented with an initial FGSI score of 7, which statistical guidelines associate with a high probability of survival, correlating with the successful outcome achieved through prompt intervention.

Urgent surgical consultation and immediate intervention are mandatory, as early, aggressive, and frequently serial debridement remains the cornerstone of definitive therapy [13]. The primary surgical objective involves wide excision of all devitalized tissue. Extensive debridement of deep fascia and muscle tissue is generally unwarranted unless direct infection is confirmed. Following aggressive initial debridement, managing the resulting extensive soft-tissue defects requires a phased surgical strategy. The implementation of Negative-Pressure Wound Therapy (NPWT), or vacuum-assisted closure, has emerged as a cornerstone in modern postoperative care [14]. NPWT significantly optimizes wound bed preparation by continuously removing exudate, reducing localized tissue edema, stimulating angiogenesis, and promoting the formation of healthy granulation tissue [14]. Once the infectious process is completely controlled and a viable wound bed is established, secondary reconstruction can be safely planned [14]. Depending on the size and location of the defect, surgical options range from direct secondary closure to advanced reconstructive techniques, including split-thickness skin grafts or local tissue flaps, which are critical to restoring functional and cosmetic integrity to the perineal and abdominal regions [14]. Current empirical antimicrobial approaches favor a triple-drug regimen, typically comprising penicillin, metronidazole, and a third-generation cephalosporin, with or without the addition of aminoglycosides. This initial antibiotic protocol must be tailored once definitive culture and sensitivity data are available [1].

Adjuvant hyperbaric oxygen therapy (HBOT) may be considered to exploit its bactericidal and bacteriostatic properties, particularly during the postoperative management of Fournier's gangrene. However, HBOT should not be given priority in the acute phase of care, where immediate operative management remains the most critical task. It must never delay or replace prompt surgical debridement [15,16].

## Conclusions

This case highlights the significance of recognizing Fournier's gangrene as a potential complication of an intra-abdominal septic process. It emphasizes the importance of considering an abdominal origin such as perforated appendicitis even when clinical signs are predominantly localized to the perineum. Early identification and the prompt initiation of aggressive medical and surgical interventions, including broad-spectrum antibiotics and thorough debridement, are crucial for achieving favorable outcomes. Adequate resuscitation and definitive source control remain the cornerstones of management to improve patient prognosis in these life-threatening scenarios.
